# Visualization and Analysis of Gene Expression in Stanford Type A Aortic Dissection Tissue Section by Spatial Transcriptomics

**DOI:** 10.3389/fgene.2021.698124

**Published:** 2021-06-28

**Authors:** Yan-Hong Li, Ying Cao, Fen Liu, Qian Zhao, Dilare Adi, Qiang Huo, Zheng Liu, Jun-Yi Luo, Bin-Bin Fang, Ting Tian, Xiao-Mei Li, Di Liu, Yi-Ning Yang

**Affiliations:** ^1^Department of Cardiology, First Affiliated Hospital of Xinjiang Medical University, Urumqi, China; ^2^Department of Clinical Laboratory, First Affiliated Hospital of Xinjiang Medical University, Urumqi, China; ^3^State Key Laboratory of Pathogenesis, Prevention and Treatment of High Incidence Diseases in Central Asian, Department of Cardiology, First Affiliated Hospital of Xinjiang Medical University, Urumqi, China; ^4^Computational Virology Group, Center for Bacteria and Virus Resources and Application, Wuhan Institute of Virology, Chinese Academy of Sciences, Wuhan, China; ^5^CAS Key Laboratory of Pathogenic Microbiology and Immunology, Institute of Microbiology, Chinese Academy of Sciences, Beijing, China; ^6^University of Chinese Academy of Sciences, Beijing, China; ^7^College of Animal Sciences and Veterinary Medicine, Guangxi University, Nanning, China; ^8^State Key Laboratory of Pathogenesis, Prevention and Treatment of High Incidence Diseases in Central Asia, First Affiliated Hospital of Xinjiang Medical University, Urumqi, China; ^9^Department of Cardiac Surgery, First Affiliated Hospital of Xinjiang Medical University, Urumqi, China; ^10^Xinjiang Medical University, Urumqi, China; ^11^People’s Hospital of Xinjiang Uygur Autonomous Region, Urumqi, China

**Keywords:** spatial transcriptomics, aortic, Stanford type A aortic dissection, gene expression, bioinformatics

## Abstract

**Background:** Spatial transcriptomics enables gene expression events to be pinpointed to a specific location in biological tissues. We developed a molecular approach for low-cell and high-fiber Stanford type A aortic dissection and preliminarily explored and visualized the heterogeneity of ascending aortic types and mapping cell-type-specific gene expression to specific anatomical domains.

**Methods:** We collected aortic samples from 15 patients with Stanford type A aortic dissection and a case of ascending aorta was randomly selected followed by 10x Genomics and spatial transcriptomics sequencing. In data processing of normalization, component analysis and dimensionality reduction analysis, different algorithms were compared to establish the pipeline suitable for human aortic tissue.

**Results:** We identified 19,879 genes based on the count level of gene expression at different locations and they were divided into seven groups based on gene expression trends. Major cell that the population may contain are indicated, and we can find different main distribution of different cell types, among which the tearing sites were mainly macrophages and stem cells. The gene expression of these different locations and the cell types they may contain are correlated and discussed in terms of their involvement in immunity, regulation of oxygen homeostasis, regulation of cell structure and basic function.

**Conclusion:** This approach provides a spatially resolved transcriptome− and tissue-wide perspective of the adult human aorta and will allow the application of human fibrous aortic tissues without any effect on genes in different layers with low RNA expression levels. Our findings will pave the way toward both a better understanding of Stanford type A aortic dissection pathogenesis and heterogeneity and the implementation of more effective personalized therapeutic approaches.

## Introduction

Stanford type A aortic dissection (AAD) is the most common thoracic aortic disease, which has a high degree of morbidity and leads to extensive medical expenditure for survivors. It may rapidly fatal if not diagnosed early and managed appropriately (Guo et al., 2016). From the biomechanical viewpoint, the mechanism of injury is based on the inability of the vascular wall to withstand high shear stress that penetrates the intimal vessel layer, resulting in blood flow to the intimal and medial layers or disruption of the media layer (Yang et al., 2020). The pathological features of aortic tissue are characterized by an enlarged and degenerative medial layer, loss or dysfunction of vascular smooth muscle cells (VSMCs), proteoglycan accumulation, and collagen and elastic fiber cross-linked disorder and fragmentation (Oller et al., 2017). The risk factors associated with the occurrence and development of AAD include hypertension, dyslipidemia, atherosclerosis, cigarette smoking, and male gender (Yang et al., 2020). Although the major aortic cell types in the whole aorta are well known (Dobnikar et al., 2018; Kalluri et al., 2019), the heterogeneity and relative contribution of different vascular cells in AAD are poorly understood.

Previous studies have demonstrated the reason for the tear during aortic dissection by regular transcriptome analyses off multiple pathways (Huang et al., 2018; Yang et al., 2018; Wang et al., 2019). However, these studies have certain limitations in accuracy. Based on single cell RNA sequencing (scRNA-seq), we can only determine the average gene expression of the ruptured tissue, and many details are lost. Identification of aortic cell-type composition depends on anatomy, and methods such as radiography and pathology may affect the reliability of the results. How the cells of the intima, media, and adventitia are affected and the role they play in the occurrence and development of the disease need to be studied urgently using novel techniques.

Spatial transcriptomics (ST) is an approach that allows the visualization and quantitative analysis of the transcriptome with spatial resolution in individual tissue samples (Stahl et al., 2016). By placing tissue sections on glass slides with arrayed oligonucleotides containing positional barcodes, high-quality cDNA libraries can be generated with precise positional information for RNA sequencing. ST has been used to study the mouse olfactory bulb (Stahl et al., 2016), breast cancer (He et al., 2020), adult human heart tissues (Asp and Salmen, 2017), melanoma tissues (Thrane et al., 2018), prostate cancer tissues (Berglund et al., 2018), gingival tissues (Lundmark et al., 2018), mouse and human spinal cord tissues (Maniatis et al., 2019), and model plant species (Giacomello et al., 2017). Asp et al. (2019) used ST to reveal the comprehensive transcriptional landscape of cell types populating the embryonic heart at three developmental stages and mapped cell type-specific gene expression to specific anatomical domains. They identified unique gene profiles that corresponded to distinct anatomical regions in each developmental stage using ST (Asp et al., 2019). High-resolution spatial heterogeneity can be captured, and the rich spatial information regarding unbiased gene expression for cells and tissues can be retained in ST results, compared with results of regular transcriptome analyses using bulk sequencing or scRNA-seq (Gerlinger et al., 2012). The heterogeneity of gene expression and spatial organization in the aorta may help identify the underlying pathogenesis of aortic dissection. Considering the particularity of aortic structure and cell composition, there is still no suitable bioinformatics algorithm to analyze the ST sequencing data of the aorta.

Here, for the first time, we provided an algorithm suitable for aortic tissue and analyzed aortic tears simultaneously at the tissue- and transcriptome-wide scales using the ST, which allowed for the identification and spatial mapping of distinct cell types, subpopulations, and cell states within heterogeneous samples. We identified the top 20 spatially related genes and identified major cell types, including smooth muscle cells (SMCs), fibroblasts, endothelial cells (ECs), and infiltrated immune cells (including macrophages, B cells, T cells, and dendritic cells). Further analysis showed that different types of cells showed different enrichment of signal pathways. For example, cluster M4 was mainly composed of macrophages and Kupffer cells, and the signaling pathways were mainly related to immunity and apoptosis. The establishment of these profiles is the first step toward obtaining an unbiased view of aortic dissection and can serve as a reference for future studies on AAD.

## Materials and Methods

### Ethics

AAD participants gave written informed consent, permission for tissue analyses, and consent for the collection of relevant clinical data before enrolling in the study as approved by the ethics committee at the First Affiliated Hospital of Xinjiang Medical University (Urumqi, China) (20150006-8). All procedures were conformed to the principles outlined in the Declaration of Helsinki.

### Participants

We recruited 15 adult patients with AAD (along with their demographic information such as age, sex, etc.) admitted to the First Affiliated Hospital of Xinjiang Medical University (Urumqi, China) from September 1, 2019, to July 1, 2020. The patients were diagnosed through history, findings of physical examination, and imaging findings according to currently accepted standards (Erbel et al., 2014). Patients were excluded if they had Marfan syndrome, Ehlers Danlos syndrome, Loeys-Dietz syndrome, Turner syndrome, congenital bi-leaflet aortic valves, aortic aneurysm, traumatic dissection, or other connective tissue disorders, and those aged < 18 years.

### Collection and Preparation of Aortic Tissue

The aortic sample was rapidly collected within 30 min after excision. The specimen was rinsed at least five times in precooled saline; then, the thrombus and redundant tissues were removed immediately using eye scissors and sterile tweezers on a clean Petri dish (placed on dry ice). Tissue samples were then sliced into approximately 6 × 6 mm sections, embedded in optimal cutting temperature compound (OCT, Sakura #4532), and frozen in isopentane (2-methylbutane, Sigma, 270342), followed by storage in liquid nitrogen for further use. The entire procedure was performed within 10 min. The fresh snap-frozen dissection aortic tissue was cryosectioned cut vertically (10 μm) using a Leica CM1950 cryostat (Leica, 14047742459) at –10°C. Typically, we should ensure the reproducibility of the same type and good quality of tissue morphology. RIN should be ≥7 and RNA quality assessment should be done before placing the tissue sections on visium spatial slides (Supplementary Figure 1).

### Preparation of Quality Control Slide

The reagent kits included visium spatial tissue optimization slides and visium spatial gene expression slides, which were used for tissue optimization and spatial gene expression, respectively. For quality control experiments, poly-T20VN oligonucleotides (IDT) were uniformly spread onto Code link-activated microscopic glass slides according to the manufacturer’s instructions (Stahl et al., 2016; Giacomello et al., 2017). Visium spatial tissue optimization slides contained eight capture mRNA areas with oligonucleotides, and each capture area was defined by an etched frame. Each probe had poly (dT) primers to allow the production of cDNA from polyadenylated mRNA. These probes did not contain a spatial barcode. The visium spatial gene expression slide had four capture areas (6.5 × 6.5 mm), each defined by a fiducial frame (fiducial frame + capture area is 8 × 8 mm). Every capture area contained ∼5,000 gene expression spots of RT-primers with unique barcode sequences. Each spot had a diameter of 50 μm (corresponding to a tissue domain). The center-to-center distance was 100 μm.

Surface primer for spatial arrays:

5′-CTACACGACGCTCTTCCGATCT-NNNNNNNNNNNNNNNN-NNNNNNNNNNNN-TTTTTTTTTTTTTTTTTTTTTTTTTTTTTTVN-3′

### Tissue Optimization (TO)

#### Fixation, Staining, and Imaging

The transported slides were placed on dry ice at –80°C and placed on a slide with tissue on a pre-warmed 37°C thermocycler adapter (10x genomics, 3000380). Then, the fixed tissues were fixed using ice-cold 100% methanol (Sigma, 34860) for 30 min and were stained with hematoxylin (Agilent, S330930-2) and eosin Y (Sigma, HT110216) (H&E) diluted 1:9 in 0.45M pH 6.0 Tris-buffer (Fisher, BP152-500) for 7 min and 1 min at room temperature, respectively. Between H&E staining, the glass slides were briefly dried, and bluing buffer (Agilent, CS70230-2) was added and washed off using RNase – and DNase – free Milli-Q water for 2 min. Then, we incubated the slide on the thermocycler adaptor with the thermal cycler (Thermo Fisher Scientific, 4375786) lid open for 5 min at 37°C and proceed to bright field imaging using a Leica SCN 400 slide scanner.

#### Tissue Permeabilization and Fluorescent cDNA Synthesis

The TO slides were placed in the slide cassette (10x genomics, 3000433) which was assembled using a slide alignment tool (10x genomics, 3000433) and was incubated with 70 μL permeabilization enzyme (10x genomics, 2000214). The positive control well includes reference RNA without any tissue. The negative control well (D2) has a tissue section not exposed to permeabilization reagents. Permeabilization times refer to the length of time (30, 24, 18, 12, 6, 3 min) tissue sections are exposed to permeabilization reagent. After permeabilization, we prepared a fluorescent reverse transcription (RT) master mix (nuclease-free water, Ambion, AM9937; RT reagent C, 10x genomics, 2000215; template switch oligo, 10x genomics, 3000228; reducing agent B, 10x genomics, 2000087; RT enzyme D, 10x genomics, 20000216) on ice according to manufacturer’s instructions, and placed a thermocycler adaptor in the thermal cycler 45 min for fluorescent cDNA synthesis.

The template switch oligo was as follows:

5′-AAGCAGTGGTATCAACGCAGAGTACATrGrGrG-3′

#### Tissue Removal and Slide Imaging

Tissue removal was performed using a tissue removal mix (tissue removal buffer, 10x genomics, 2000221, tissue removal enzyme, 10x genomics, 3000387) which was incubated in the thermal cycler based on protocol. Then, we removed the slide from the slide cassette and centrifuged it for 30 s in a slide spinner. Fluorescence imaging was performed to all captures areas together under the same fluorescence settings using a Leica DMi8 fluorescence microscope.

### Visium Spatial Gene Expression

#### Fixation, Staining, Imaging, Permeabilization, and RT

The sections were fixed, stained, and bright field imaging was performed as described previously; the process of tissue removal was skipped. Next, we added the permeabilization enzyme on top of the tissue, which was determined by the optimization conditions. RT mixtures used for spatial arrays (intended for library preparation and sequencing) were different from the RT mixtures used for the optimization of spatial arrays (intended for library preparation and sequencing). Then, the second strand mix was prepared (second strand reagent, 10x genomics, 2000219; second strand primer, 10x genomics, 2000217; second Strand enzyme, 10x genomics, 2000218) on ice and added to the slide incubated on the thermal cycler. Subsequently, 0.08M KOH (Sigma, 1002868722) was added to denature the second strand, which was then collected. To determine the number of PCR cycles required for indexing, 1 μL of the purified cDNA was mixed with 9 μL of qPCR mixtures (KAPA SYBR FAST qPCR master mix, KAPA Biosystems, KK4600; cDNA primers, 10x genomics, 2000089). Then, qPCR amplifications were performed using a qPCR instrument (Applied Biosystems, 4471087), followed by cDNA amplification and quality control, purification, and transfer to separate tubes. After capturing and reverse-transcribing mRNA, we constructed a spatial gene expression library.

The second strand primer was as follows:

5′-AAGCAGTGGTATCAACGCAGAG-3′

cDNA primers:

Forward primer: 5′-CTACACGACGCTCTTCCGATCT-3′

Reverse Primer: 5′-AAGCAGTGGTATCAACGCAGAG-3′

#### Spatial Library Construction and Sequencing

A spatial library was prepared with 10x genomics following the user guide provided. First, fragmentation, end repair, and A-tailing were performed. The obtained cDNA profile could vary; thus, a fragmentation mix had to be prepared on ice. The amplified-cDNA was then fragmented, ligated with the adapter and sample index, and selected using SPRI beads (Beckman Coulter, B23318) to an average size of 300 bp. The quality of the libraries was evaluated at two points during the process: first, by analyzing the fragment lengths and library concentration after ligation cleanup, and second, by analyzing the library amplifiability after the final cDNA synthesis on an Agilent bioanalyzer high-sensitivity chip (Jemt et al., 2016). The constructed library was sequenced on an Illumina Nova 6000 platform.

### Processing and Mapping of ST Raw Reads

Paired end 150 bp sequencing was performed using Illumina’s NOVA 6000 platform. The library was sequenced using paired-end 150 bp paired-end reads using Illumina’s Nova 6000 platform. Following demultiplexing the Illumina sequencer’s base call files (BCLs) for each flowcell directory and converted BCLs files to FASTQ files using Bcl2Fastq2 Conversion Software (v2.20). Subsequently, the converted FASTQ file was subjected to quality control, and low-quality reads (including reads with higher N content) were filtered out. Then, the read 1 and read 2 FASTQ files were trimmed using Cutadapt (version 1.16). The read 1FASTQ file was trimmed to only the linker sequence with a length of 28 bp, and the read 2FASTQ file was only 120 bp in length; the rest files were deleted because they did not require subsequent analysis. To generate spatial feature counts for a single library using automatic fiducial alignment and tissue detection, the trimmed reads were processed with the Space Ranger pipeline (version 1.0.0) with the following arguments: “–sample V19N13_040_A1_20200728NC –slide V19N13-040 –area A1 –localcores 20 –localmem 64 –image mmexport1596289888734.jpg.”

To compare the results of automatic alignment and manual alignment, a tissue assignment json file was generated in Loupe Browser, and Space Ranger count was run with “–sample V19N13_040_A1_20200728NC –slide V19N13-040 –area A1 –localcores 20 –localmem 64 –image mmexport1596289888734.jpg –loupe-alignment V19N13-040-A1.json” arguments. The reference genome used in the two Space Ranger runs was the GRCh38 v93 genome.

### Selection of Methods for ST Data Analysis

The gene-spot matrices generated after ST data processing and Visium samples were analyzed using the Seurat package (version 3.1.3) in R (Butler et al., 2018). To explore the differences in normalization methods, SCTransform and log normalization were performed separately, another covariate was used to calculate the correlation of features that were grouped into groups using the Group Correlation function (settings: min.cells = 5, ngroups = 6), and the correlation between their results and the number of UMIs was tested. The results obtained by the normalization method with better correlation were selected for PCA and ICA. Then, the first 20, 30, and 50 elements analyzed by PCA and ICA were selected for subsequent analysis. Clustering of each spot is based on K-Nearest Neighbor algorithm. The distance from each point to other points was calculated first, and the shared nearest neighbor (SNN) graph was constructed according to the distance between sample points. Finally, the FindClusters function was used to determine the cluster (FindNeighbors settings: reduction = “pca/ica,” nn.method = “rann,” dims = 1:20/30/50, k.param = 20; Find Clusters settings: resolution = 0.8, method = “matrix,” algorithm = 1). For clustering and re-dimension-reduction through uniform manifold approximation and projection (UMAP) (Becht et al., 2018) and t-distributed stochastic neighbor embedding (t-SNE) methods (van der Maaten and Geoffrey, 2008). The two methods of dimensionality reduction were evaluated based on the clustering of spot types.

### Identification of Cluster-Specific Genes

For each cluster that was identified, the differentially expressed genes (DEGs) were determined in relation to all other spots. A spatial cluster gene list was first generated for all genes differentially expressed in ST clusters (average logFC > 0.25, adjusted *p*-value < 0.05, and only return positive genes). The mean expression of each gene was calculated across all spots in the cluster to identify genes that were enriched in a specific cluster. Each gene from one cluster was compared with the average expression of the same gene from the spots of all other clusters. The genes were ranked according to their expression differences, and the DEGs with the largest changes in each cluster were checked and visualized using heat maps.

### Identification of Cell Types

Two databases have been used to identify cell types at different levels. First, the CellMarker database (Zhang et al., 2019) was used as a reference to classify the cell subpopulations from the annotations of cluster-specific genes. All human cell types and their corresponding marker genes in the CellMarker database were downloaded and integrated into a dataset. Then, the cluster-specific genes in the sample performed a hypergeometric test on the dataset with the help of the enricher function in the clusterProfiler (version 3.12.0) (Yu et al., 2012a) (settings: *p*-value Cutoff = 0.005). The different cell types annotated for each cluster were finally determined according to their enrichment factors and artificial corrections. Then, the count of gene expression on each spot was compared with the Human Cell Landscape (HCL) database using the scHCL function in order to identify the Cell types that might be contained at different locations of the sample (Han et al., 2020) (settings: numbers_plot = 10).

### Gene Functional Annotation

For the DEGs identified in each cluster, cluster Profiler (version 3.12.0) (Yu et al., 2012b) was used to perform Gene ontology (GO) and Kyoto Encyclopedia of Genes and Genomes (KEGG) pathway annotation, which supports statistical analysis and visual expression of the functions of genes and gene clusters. The Cluster Profiler package provides enriched GO and enriched KEGG functions to perform enrichment tests for gene ontology terms and KEGG biological pathways based on hypergeometric distribution. To reduce the false discovery rate in multiple testing, we chose FDR-corrected *p*-value less than 0.05 as the threshold.

### Multi-Color Immunofluorescence Staining

The tissue was collected and prepared for the OCT-embedded frozen tissues and 8-mm-thick serial sections were prepared for. The confirmation of cell types was analyzed using Opal 7-Colour Manual IHC Kit (PerkinElmer, United States) according to the manufacturer’s protocol. In brief, antigen was retrieved by AR9 buffer (pH 6.0, PerkinElmer, United States) and boiled in the oven for 15 min. After a pre-incubation with blocking buffer at room temperature for 10 min, the sections were incubated at room temperature for 1 h with mouse anti-human CD31 (Abcam 9498, United Kingdom, 1:200), rabbit anti-human CD163 (Abcam 9519, United Kingdom, 1:1000), rabbit anti-human CALD1 (Abcam 32330, United Kingdom, 1:300), rabbit anti-human HLA-DR (Abcam 92511, United Kingdom, 1:100), rabbit anti-human ACTA2 (Abcam 124964, United Kingdom, 1:300), and mouse anti-human ELN (Abcam 9519, United Kingdom, 1:100). A secondary horseradish peroxidase-conjugated antibody (PerkinElmer, United States) were added and incubated at room temperature for 10 min. Signal amplification was performed using TSA working solution diluted at 1:100 in 1 × amplification diluent (PerkinElmer, United States) and incubated at room temperature for 10 min. The other validations by multi-color IHC were performed using the same protocols with different primary antibodies as follows. The multispectral imaging was collected by Mantra Quantitative Pathology Workstation (PerkinElmer, CLS140089) at 20 × magnification and analyzed by In Form Advanced Image Analysis Software (PerkinElmer) version 2.3. For each section, a total of 5–10 high-power fields were taken based on their tissue sizes.

## Results

### Patient and Tissue Spatial Gene Expression Information

**FIGURE 1:**
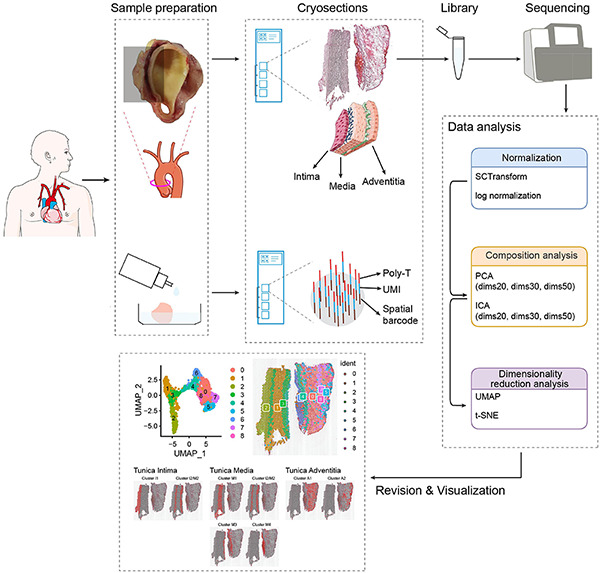
Study design for ST in aortic dissection. Tissue of aortic dissection from the patient was dissociated and embedded in OCT; a fresh thin tissue section was obtained from cryosection, which was attached and fixed to each spot on the microarray slide; the library was sequenced and further processed to map the expressed genes to the spatial locations at which they were expressed; establishment of data analysis methods and visualization of cell populations.

We randomly selected one AAD patient (involving ascending aortic) with hypertension (male, 50 years old) from the first affiliated hospital of Xinjiang Medical University, who was well characterized and had a typical phenotype of AAD based on computed tomography angiography (CTA) results. The demographic data, operative details, and microarray data of the tissue are presented in Supplementary Table 1. Supplementary Table 1 summarizes the data of the patient. Overall, 19,879 genes within 1,873 spot regions were analyzed in one tissue section, yielding a mean of 181,097 reads/spot with median gene and median unique molecular identifier (UMI) counts of 2514, respectively. The number of cells located within the tissue domain (each spot with a diameter of 50 μm) is estimated range from 3–10 for aortic section depending on if cells are longitudinal- or cross-sectioned. Longitudinally oriented aortic cells can potentially cover more than one single feature and numerous features contain different cell types such as ECs, SMCs, and fibroblasts. A schematic diagram of the experimental design and data analysis is shown in Figure 1. We compared different algorithms for the steps of normalization, component analysis, and dimensionality reduction analysis. According to the final cell annotation results, we evaluated the algorithm combination and created a pipeline suitable for analysis of human aortic tissues.

### Quality Control and ST Sequencing Data Analysis in Aortic Dissection Tissue

**FIGURE 2:**
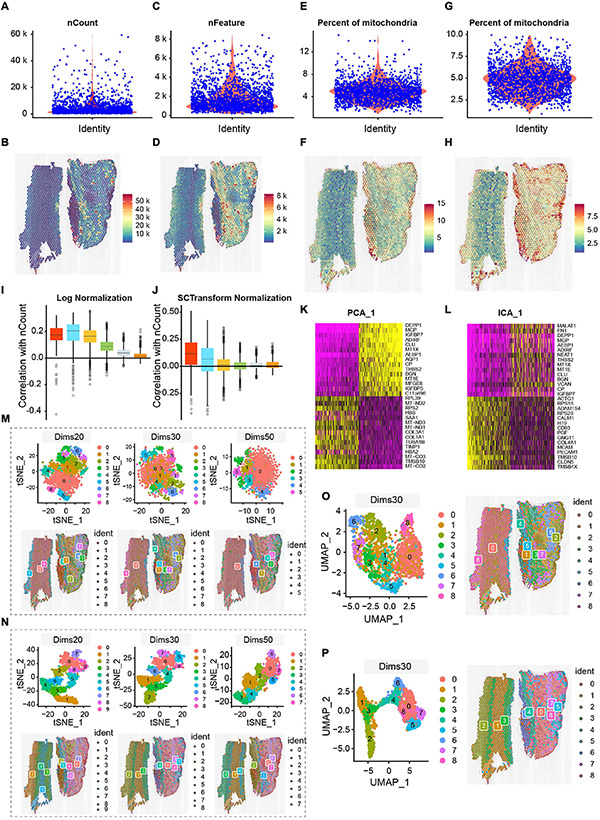
Quality control and ST data analysis. **(A,B)** The number of nCount_RNA is range of 10,000–20,000, with the maximum not exceeding 60,000, and spatial UMIs distribution is concentrated in the aortic of tunica media and external. **(C,D)** The number of genes is mostly between 1,000 and 7,500. Combined with the distribution of UMIs data, the region with a high number of genes also had a high number of UMIs. **(E,F)** The percentage of mitochondria is low, between 1 and 12%. Correspondingly, the distribution of spatial UMIs in the tunica media and external is also rare. **(G,H)** Cells with >10% mitochondrial reads are filtered, and display distribution of spatial UMIs. The colors from blue to red represented increasing number of expression. **(I,J)** Comparison of normalization methods (log and SCTransform normalization), the SCTransform normalization is superior to Log Normalization. **(K,L)** Comparison of compositional analysis (PCA and ICA). **(M–P)** Comparison of dimensionality reduction and clustering methods, among them, **(M,O)** are under the ICA condition, the distribution of t-SNE (dims 20, 30, 50) and UMAP (dims 30); **(N,P)** are under the PCA condition, the distribution of t-SNE (dims 20, 30, 50) and UMAP (dims 30). Overall, PCA dims 30 combined with UMAP dimensionality reduction cluster analysis is an appropriate method. The Clusters are labeled using different colors.

We chose three indicators—the count of RNA, count of genes, and percentage of mitochondrial genes (nCount_RNA, nFeature_RNA, and percent mitochondrial)—to demonstrate the reliability of data; the spatial UMIs and gene distribution are shown in Figures 2A–H. Among them, some cells with >10% mitochondrial reads were filtered, and dead cells were removed (Ji et al., 2020; Figures 2G,H). The correlation between UMIs and genes obtained by two different normalization methods—SCTransform normalization and log normalization. The box plot of genes was divided into six groups according to their average expression levels (Figures 2I,J), which indicated that the SCTransform normalization method was better for fully normalizing highly expressed genes. Similarly, we applied two widely employed component analysis methods—principal component analysis (PCA) and independent component analysis (ICA)—for the first dimensionality reduction analysis. The first principal component heat maps of the top 30 genes obtained by PCA and ICA are shown in Figures 2K,L. We observed that only 12 genes were found to be co-expressed by the first principal component of the two methods, indicating that PCA and ICA provided significantly different results. The merits and demerits of two component analysis methods cannot be assessed at the genetic level alone. Therefore, the results of the two component analysis methods were selected for t-SNE and UMAP dimensionality reduction analysis. We also compared the effect of dimension selection of component analysis on the results by selecting the top 20, 30, and 50 components. Finally, the optimal combination of the component analysis method and the dimensionality reduction algorithm was determined according to annotation of cell type. ICA results are shown in Figure 2M, and the first 20, 30, and 50 principal components were selected for t-SNE dimensionality reduction processing. The first row is the t-SNE clustering result, and the second row provides a visualization of the corresponding position of the clustering result on the tissue. Dims 20, 30, and 50 were divided into 10, 9, and 8 clusters after dimensionality reduction, respectively. The smaller the number of components selected, the more clusters were obtained on the spots. The results of t-SNE dimensionality reduction analysis of PCA are shown in Figure 2N. ICA and PCA provided the same number of clusters when the number of dims was confirmed. However, we found that PCA exhibited less overlap and better cluster independence than ICA in the visualization results. Similarly, we also selected different numbers of dims for UMAP clustering analysis of the two component analysis methods. The results of dims 30 are shown in Figures 2O,P. The UMAP dimensionality reduction methods of dims 20 and dims 50 are shown in Supplementary Figure 2. The visualization results of t-SNE may exaggerate the differences between cell populations and ignore the potential associations between these cell populations (Figures 2N,P). Subsequent cell type annotation results also showed that the effect obtained by PCA dims 30 combined with UMAP dimensionality reduction cluster analysis was more consistent with the actual cell type distribution. The high quality of data guaranteed cell- and gene-level downstream analysis.

### Aortic Tissue ST Sequencing Identifies Spatial Locations of Genes in the Human Aorta

**FIGURE 3:**
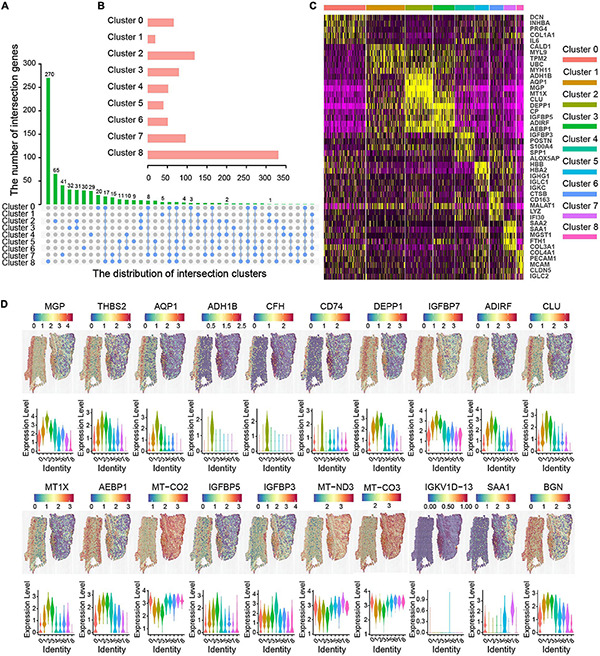
Molecular characteristics related to spatial location in ascending aortic dissection tissue. **(A)** Correlation clusters of overlapping DEGs. The column length represents the number of overlapping genes. **(B)** The bar chart represents the number of genes in each cluster. **(C)** The heat map displays the top five (by average log [fold change]) genes in each cluster. The *X*-axis represents the distribution of intersection clusters; *Y*-axis represents the number of intersection genes. **(D)** The top 20 genes of location and expression level were in each layer. Violin plots of gene expression levels show different clusters with different colors.

The PCA combined dimensional reduction method of UMAP was performed and the first 30 principal components were annotated to obtain nine clusters in three layers of the aortic sections. We analyzed the intersection of significant genes among different clusters and found that the number of intersection genes were relatively small (Figure 3A). The number of genes expression in each cluster is shown in Figure 3B. The results showed that cluster 8 had the highest number of significant genes, followed by cluster 2; clusters 4 and 6 had a similar counts of significant genes, and cluster 1 had the lowest number of significant gene (Figure 3B). The top five DEG of these clusters, according to the detected mRNA transcript amounts, are displayed in a heat map (Figure 3C). Genes with significantly different expression were used for cell annotation. Then, we displayed the top 20 genes that were spatially related in AAD aortic tissue, as shown in Figure 3D. Among these spatially related genes, six genes (MGP, THBS2, AQP1, ADH1B, CFH, and CD74) were highest expressed in the intima, which are associated with the regulation of human ECs calcification and inflammation. DEPP1, IGFBP7, ADIRF, CLU, MT1X, and AEBP1 were highly expressed in both the tunica intima and media, and the proteins encoded by these genes may play a significant role in smooth muscle cell differentiation, migration, and apoptosis. Four genes (MT-CO2, MT-CO3, IGFBP5, and IGFBP3) were highly expressed in both the tunica media and adventitia, which have role in stem cell differentiation. Three genes (IGKV1D-13, SAA1, and BGN) were highly expressed in the adventitia, in which high levels of the proteins are associated with inflammatory diseases. One gene (MT-ND3) was highly expressed in the three layers of the ascending aorta; this gene may act as a transcriptional regulator for numerous genes, including some genes involved in cell metastasis and migration, and are involved in cell cycle regulation.

### Distribution of Cell Types at Different Location

**FIGURE 4:**
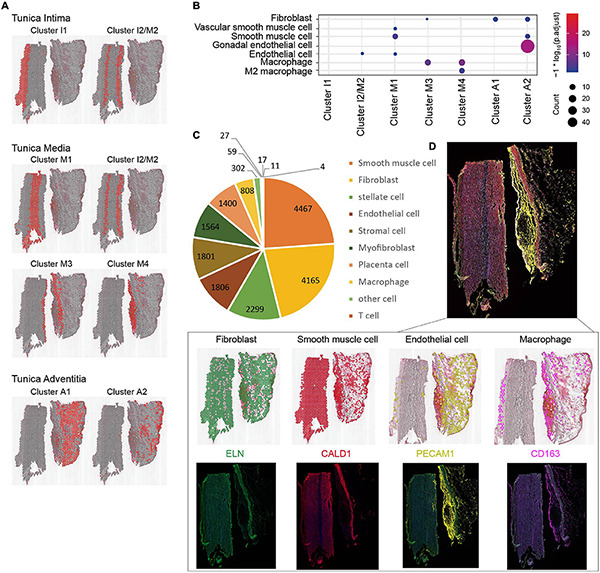
Cell types identification of aortic dissection tissue. **(A)** The spatial location distribution of cell types in each cluster. Red region represents each cluster. **(B)** Major cell types by CellMarker database. **(C)** The major cell types and its number calculated by HCL database. **(D)** Verify the accuracy of cell types by multi-color immunofluorescence.

In order to determine cell types, we combined CellMarker and HCL database to annotate the data and compare the obtained cell types. All human cell types and marker genes in the CellMarker database were downloaded as the basis for cell identification. Cell types identified by CellMarker database are shown in Supplementary Figure 3; the genes for each cluster are listed in the Supplementary Table 2, and the position of cluster is displayed in Figure 4A. We found that cluster 0 and cluster 7 cell types were very similar, and cluster 5 and cluster 8 cell types were very similar. Then, the 9 clusters were merged into 7 clusters and renamed them as cluster intima (clusters I1 and I2/M2), cluster media (clusters I2/M2, M1, M3, and M4), and cluster adventitia (clusters A1 and A2) by manual annotation. Subsequently, the gene expression count on each spot was compared with the HCL database and the distribution and score of possible cell types on the spot were obtained. The major cell types (ECs, SMCs, fibroblast, and immune cells) are shown in Figure 4B. We found ECs located in cluster I2/M2 on the tunica intima layer, SMCs, fibroblast and macrophage located in the tunica media layer (clusters M1, M3, and M4), and ECs, fibroblast located in clusters A1 and A2 on the tunica adventitia layer. According to the HCL database, we identified the cell types and calculated its number, and found the ECs, SMCs, fibroblast, and immune cells account for a large proportion (Figure 4C), which was consistent with the CellMarker database. The accuracy of cell type identification was further confirmed by multi-color immunofluorescence (Figure 4D). The function of cell types in AAD can be inferred: cluster I1 and cluster I2/M2 cells both displayed a high correlation with differentiation, regeneration, and nerve conduction functions, such as progenitor cells, astrocytes, and microglial cells, and so on. Cells from cluster I2/M2, M1, M3, and M4 showed a high level of support and immune function, which were located in the tear position. Clusters M1 and M2 also contained numerous fibrous cells and Leydig cells, which maintain the structure of the aorta. We identified numerous types of stem cells and progenitor cells, which were closely related to vascular remodeling in clusters I1 and A1.

### GO and KEGG Analysis of DEGs in Spatial Expression

**FIGURE 5:**
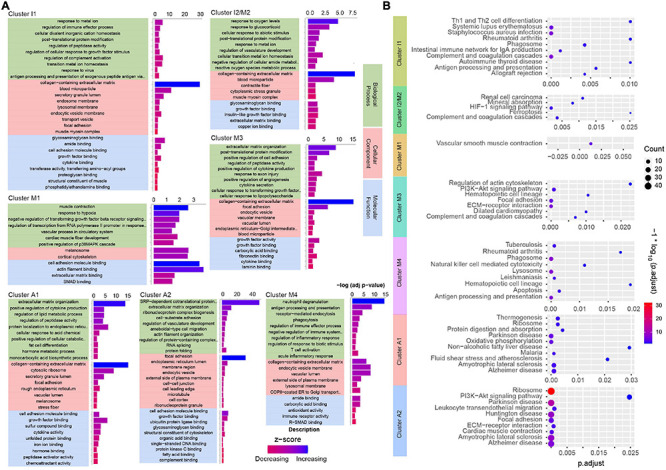
Analysis of GO and KEGG enrichment of clusters in ascending aortic dissection tissue. **(A)** Analysis of genes enriched by GO terms (biological processes, cellular component, molecular function) (p. adjust) in each cluster. The function is reflected by *z*-score and up color-coded from red to blue. **(B)** KEGG analysis for hallmark genes of enriched pathways in each cluster. The gradient color represents the *P*-value; the size of the black spots represents the gene number.

After confirming the cell types in the pathological state, we next applied bioinformatics tools to determine the biological pathways affected by type A aortic dissection. We used GO gene annotation to identify cellular components and biological signals that were correlated with the spatial location in each cluster (Figure 5A). Pathway enrichment analysis was performed using KEGG to annotate the function (Figure 5B). The GO and KEGG function annotation results indicated that different types of cells at different locations showed different functional enrichment of signal pathways. For instance, cluster I1 and cluster I2/M2 were distributed in the tunica intima, which were involved in the regulation of oxygen levels and cellular activity in biological process. KEGG analysis enriched several pathways involved in the oxygen regulation of cell cycle activities and immunity moderation in the two clusters. We observed that the genes in cluster M1 and cluster I2/M2 in tunica media regulated muscle contraction and extracellular matrix activity. Similarly, pathways (vascular smooth muscle contraction, ECM-receptor interaction) enriched by KEGG were related to a highly specialized cell whose principal function was contraction. Cells in cluster M3 and cluster M4 were located in the tunica media of the tear, and involved numerous immune cells such as neutrophils, and T cells and so on in biological process. Interestingly, KEGG also enriched some pathways related to antigen processing and presentation, apoptosis, and focal adhesion, which play essential roles in cell motility, cell proliferation, cell differentiation, regulation of gene expression, and cell survival. GO analysis showed that the genes involved in neuropathic diseases and vascular functions were present in clusters A1 and A2. Likewise, genes involved in several neuroregulatory pathways that contribute to neuron degeneration, mitochondrial dysfunction, and oxidative stress in the tunica adventitia were observed by KEGG enrichment analysis. In summary, the GO database screened for genes involved in biological processes in cells, and their functions were completely consistent with KEGG enriched pathways.

### Visualized Gene Expression Patterns in Aortic Dissection Tissue

**FIGURE 6:**
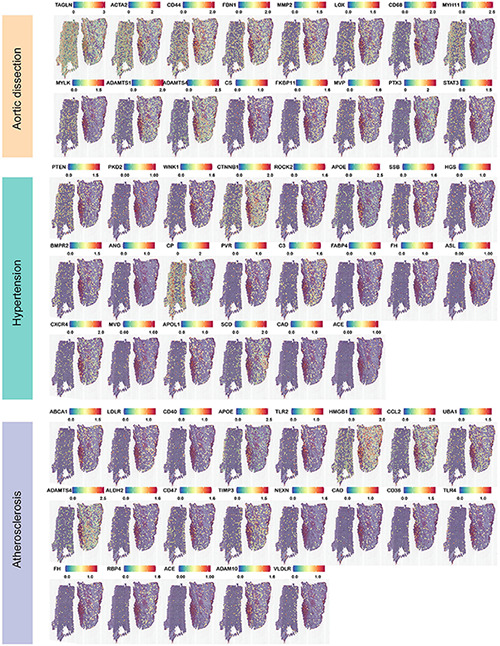
Expression of highly expressed genes in dissection-related pathogenic factors in aortic dissection. Aortic dissection has reported the expression of highly expressed genes (hypertension, atherosclerosis) in dissection tissues of pathogenic factors. ST profiles of hypertension and atherosclerosis are listed.

We retrieved 30 genes related to aortic dissection, and displayed the top 16 genes closely related to location information based on gene expression higher than 1.5, which are shown in Figure 6. The visual gene expression profile shows that, six genes were highly expressed in three layers—*TAGLN*, *ACTA2*, *CD44*, *FBN1*, *MMP2*, and *LOX*; three genes were highly expressed in the tunica intima and media—*CD68*, *MYH11*, and *MYLK*; and seven genes were highly expressed in the tunica adventitia—*ADAMTS1*, *ADAMTS4*, *CS*, *FKBP11*, *MVP*, *PTX3*, and *STAT3*. The genes closely associated with the pathogenesis of aortic dissection were also searched (Akutsu, 2019), and the aortic dissection tissue presents top 22 and top 21 genes of the location information based on the gene expression higher than 1.0 in Figure 6, respectively. We found that genes related to hypertension—such as *ACE*, *ANG*, *CAD*, *BMPR2*, and other genes—were also significantly expressed in ascending aortic dissection tissue reported in the literature. Among these genes, expressed in all three layers of the aorta were *PTEN*, *PKD2*, *WNK1*, *CTNNB1*, *ROCK2*, *APOE*, *SSB*, *HGS*, and *BMPR2*. The genes expressed in the tunica intima and media of the aorta were *CP*, *PVR*, and *C3*. The genes expressed in the tunica adventitia of the aorta were *FABP4*, *FH*, *ASL*, *CXCR4*, *MVD*, *APOL1*, *SCD*, *CAD*, and *ACE*. Medical history was reviewed and showed that the patient had hypertension upon admission. We also found that atherosclerotic genes were expressed in the three layers of the ascending aorta: *ABCA1*, *LDLR*, *CD40*, *APOE*, *TLR2*, *HMGB1*, *CCL2*, *UBA1*, and *ADAMTS4*; genes that were highly expressed in the tunica intima and medial were *ALDH2*, *CD47*, *TIMP3*, and *NEXN*, and genes that were highly expressed in the tunica adventitia were *CAD*, *CD36*, *TLR4*, *FH*, *RBP4*, *ACE*, *ADAM10*, and *VLDLR*. Combined with the patient’s history, we found that hypertension did cause aortic dissection, and atherosclerosis was an important risk factor for aortic dissection, a result consistent with those of previous studies. However, genes associated with diabetes, inflammation, oxidative stress, and dyslipidemia were less expressed in the aortic dissection. The genes position information of these disease-causing factors in the aortic dissection tissue is listed in the Supplementary Figure 4.

## Discussion

AAD is a severe vascular disease with high mortality and limited therapeutic options (Nienaber and Clough, 2015). Understanding the biological functions, networks, and interactions of the different cell types that regulate aortic and AAD development requires both cellular information and a spatial context (Asp et al., 2019). Consequently, a visium spatial gene expression solution has been proposed to the study human aortic dissection. Here, for the first time, we provided a pipeline for aortic tissue separation and data quality control of aortic cell types through ST and showed that the pipeline can be applied to human fibrous aortic tissues with low RNA expression levels in different layers. We also preliminarily depicted a molecular landscape for ascending aortic dissection of the three layers of the aorta. Furthermore, we displayed the positional information of genes related to pathogenic factors in the aortic tissue and elaborated on the expression patterns of signal pathways in different aortic cell clusters.

The most challenging issue with the visium spatial gene expression solution is the total RNA extraction and fluorescence capture of the ascending aorta tissue (compared with other human tissue types) because the vascular tissue contains a low density of cells and a large proportion of fibrous tissue, due to which performing experiments becomes difficult. Rigorous precautions must be taken to avoid degradation of RNA during its dissociation, thereby impairing both RNA quality and yield. A further complication is that in standard RNA-seq, whole tissue biopsies are homogenized and average representations of expression profiles within the entire sample are obtained. Consequently, information on spatial patterns of gene expression is lost and signals from subpopulations of cells with deviant profiles, such as those with low-level gene expression in the tear and dysfunctional tunica medial, are obscured. To overcome these deficiencies, we aimed to analyze the gene expression in different layers of human AAD tissues using a novel ST sequencing, which allows more refined analysis of gene expression in a tissue section. We covered 1,873 spot, detected 19,879 genes, and simultaneously associated gene expression with specific cell types.

Besides, different computational methods were compared in our analysis process to identify the best processing pipeline. To our knowledge, because of the high dimensionality of ST data, differences in gene length and genome coverage, and experimental errors in processes such as cell lysis and RT, the standardization of preliminary data is critical to the interpretation of subsequent analysis results. Figures 2I,J show the correlation between each gene and the number of UMIs. We grouped the genes according to their mean expression and boxplots of these correlations. Log-normalization failed to adequately normalize the genes in the first four groups, which indicates that technical factors continue to affect the normalized expression estimates of highly expressed genes. On the contrary, SCTransform normalization substantially reduces this effect. The normalized data were analyzed by PCA and ICA. PCA assumes that the original components are unrelated to one another and orthogonal, and ICA assumes that the original components are independent of one another. Both were used to identify the cell types contained in the populations. To avoid overcrowding among clusters and obtain the optimal cell clustering, PCA and ICA dimension-reduction should be performed by clustering and re-dimension-reduction analysis using t-SNE and UMAP algorithms. Figures 2N,P show that the UMAP algorithm retains more global structures than t-SNE, especially the continuity between cell subsets. The specific pipeline enables the aortic histomorphology to map the corresponding spatial location more effectively according to the results of cell annotation.

The artery includes an abundance of multifunctional cell populations, with each of them distinctly involved in cardiovascular diseases, such as atherosclerosis and aortic dissection. Visium spatial gene expression solutions cannot reach the resolution of a single cell, which is an inevitable technical problem. We can only classify genes according to the gene expression pattern, and then describe the cells that may be contained in each group according to the existing marker genes. Therefore, two databases were used to identify the cell types and their accuracy was verified by multi-color immunofluorescence. We first presented an appropriate approach to visualize the spatial transcriptional atlas of cell types. Hence, individual transcriptomes received from each feature will provide spatial gene expression profiles. We then identified their heterogeneity in human ascending aortic dissection, which enabled the analysis of various cells corresponding to specific genes, location distribution, and functions in the tissue section. Various studies have used scRNA-seq to delineate the heterogeneity of vascular cells, including VSMCs (Dobnikar et al., 2018), ECs (Kalluri et al., 2019), macrophages (Cochain et al., 2018), and aortic adventitia cells (Gu et al., 2019) in healthy and diseased state of arteries. The ST sequencing data were analyzed using the combined SCTransform normalization, PCA dim 30, and UMAP dimensionality reduction clustering method to annotate cell types. We provided characteristic changes in the three major vascular cell types (vascular structural correlation cells, vascular development correlation cells, and immune cells) according to distinct functions in seven clusters in the three aortic layers. Consistently, both vascular resident cells, including SMCs, fibroblasts, and ECs, and infiltrating immune cells, including macrophages, B cells, T cells, and dendritic cells, were observed (Hadi et al., 2018). In the tunica intima, we identified many granulosa cells, microglial cells, and ECs, which were different from those in healthy aorta. This is associated with inflammatory infiltrating of the arterial intima, weakened vascular walls, degradation of the cytoplasmic matrix, and endothelial cells eliciting an immune response that regulates blood flow and recruits immune cells. There is a need for complementary research in the field to further highlight and compare the results with those from other locations in aorta. In the tunica media, the cell types identified were mainly SMCs and VSMCs, which have specialized functions of maintaining a stable vascular structure. These results are similar to those reported by Zhao et al. (2020). Based on the characterized transcriptomic profile, immune cells accounted for more than 80% of the total cells in the tear of tunica media, which might have important functions in cell activation in response to shear stress of blood pressure. Consistent with the results in the heart, healthy large blood vessels appear to have more endothelial cell heterogeneity, whereas mural cells exhibit less transcriptional variability (Chavkin and Hirschi, 2020). We also identified numerous stem cells; it is also possible that adventitial stem cells or myofibroblasts may transdifferentiate into a contractile phenotype and migrate into the tunica media (Pedroza et al., 2020). Adding another layer of diversity to the cellular landscape of tunica adventitia, gonadal endothelial cells and neuronal cells were detected by ST sequencing despite the rarity of resident macrophages, which attract immune cells. The largest population of cells was of stem cells, which control and maintain cell regeneration and play an important role in angiogenesis and remodeling (Baron et al., 2018; Brown et al., 2018).

In the diseased state, these adaptive changes do not return to baseline levels but instead initiate pathological vascular alterations observed with AAD. We revealed that DEGs included those related to cellular activity (*AEBP1*, *ADIRF*, and *IGFBP5*), inflammation (*MGP*, *BGN*, and *SAA1*), and neurons (*CLU*, *MT-CO2*, and *ADH1B*), as well as the genes and feature signaling pathways for each cluster. The KEGG pathway annotation showed that all DEGs were significantly enriched in multiple pathways, including 15 in the tunica intima (clusters I1 and I2/M2), 21 in the tunica media (clusters M1, I2/M2, M3, and M4), and 20 in the tunica adventitia (clusters A1 and A2). They play an important role in the process of VSMCs loss or dysfunction, proteoglycan accumulation, and collagen and elastic fiber cross-linked disorder and fragmentation. Additionally the results were consistent with that observed for the heterogeneity of cell type functions, which serves as a direct evidence for subsequent study. Preliminary result demonstrating the pathways suggests that AAD is among the complex mechanisms through which they participate in vascular injury repair and thus is a potentially interesting field.

There were some limitations in our study. First, as this study contains a limited number of subjects no conclusions about AAD disease progression can be made. More samples are required to elaborate potential mechanism underlying the interactions between cells and aortic dissection. Second, while ST sequencing permits simultaneous characterization of cell type within the aorta, this data provides a limited view of the true functional changes in AAD pathogenesis that are undoubtedly affected by cellular processes other locations of aorta Other positions are required to enhance the reliability of the results, including aortic arch, the left common carotid and the left subclavian artery.

## Conclusion

We provided a reliable ST sequencing data computational method available for the scientific community to further explore the key factors and pathways involved physiologically in the low cell density and high fiber of the aorta. The pipeline was applied to cell annotation and pathway enrichment analysis corresponding to cell location and our findings may provide insights into the function and regulation of AAD onset and progression and pave the way for selective targeting of causative cell populations in vascular diseases.

## Data Availability Statement

The datasets presented in this study can be found in online repositories. The names of the repository/repositories and accession number(s) can be found below: PRJNA730333.

## Ethics Statement

The studies involving human participants were reviewed and approved by the ethics committee at the First Affiliated Hospital of Xinjiang Medical University. The patients/participants provided their written informed consent to participate in this study. Written informed consent was obtained from the individual(s) for the publication of any potentially identifiable images or data included in this article.

## Author Contributions

Y-NY and DL conceived the project. Y-NY, DL, and X-ML supervised the study. QH, ZL, Y-HL, TT, J-YL, and FL collected the samples. Y-HL and YC constructed RNA-seq libraries and carried out sequencing and designed the dynamic cross-tissue network analysis method. B-BF, DA, and QZ performed the cryosectioned. YC, Y-HL, and DL performed the bioinformatics and data analysis. Y-HL and YC drafted the manuscript. DL and Y-NY revised the manuscript. All the authors read and approved the final manuscript.

## Conflict of Interest

The authors declare that the research was conducted in the absence of any commercial or financial relationships that could be construed as a potential conflict of interest.

## Supplementary Material

Supplementary Figure 1 The tissue contains RNA of good quality.

Supplementary Figure 2 Comparison of compositional analysis and dimensionality reduction. **(A)** The first row shows a comparison PCA of UMAP dim 20 and dim 50. The second row shows the UMIs distribution of each cluster, and the different clusters are labeled by using different colors. **(B)** The first row shows a comparison ICA of UMAP dim 20 and dim 50. The second row shows the UMIs distribution of each cluster, and different clusters are labeled using different colors.

Supplementary Figure 3 Cell types identified by CellMarker database. The identified cell types in each cluster, which are annotated based on existed marker gene information. The gradient color represents the *P*-value; the size of the black spots represents the gene number.

Supplementary Figure 4 Expression of highly expressed genes in dissection-related pathogenic factors in aortic dissection. Aortic dissection has reported the expression of highly expressed genes in dissection tissues of pathogenic factors (diabetes, dyslipidemia, inflammation, oxidative stress). These factors genes of ST profiles are listed.

Supplementary Table 1 Basic information of patient.

Supplementary Table 2 The genes of each cluster.
