# Supplementary figures and images for: Visualization and Analysis of Gene Expression in Stanford Type A Aortic Dissection Tissue Section by Spatial Transcriptomics
Source: Front Genet. 2021 Jun 28;12:698124. doi: 10.3389/fgene.2021.698124 (PMC8275070; doi:10.3389/fgene.2021.698124)

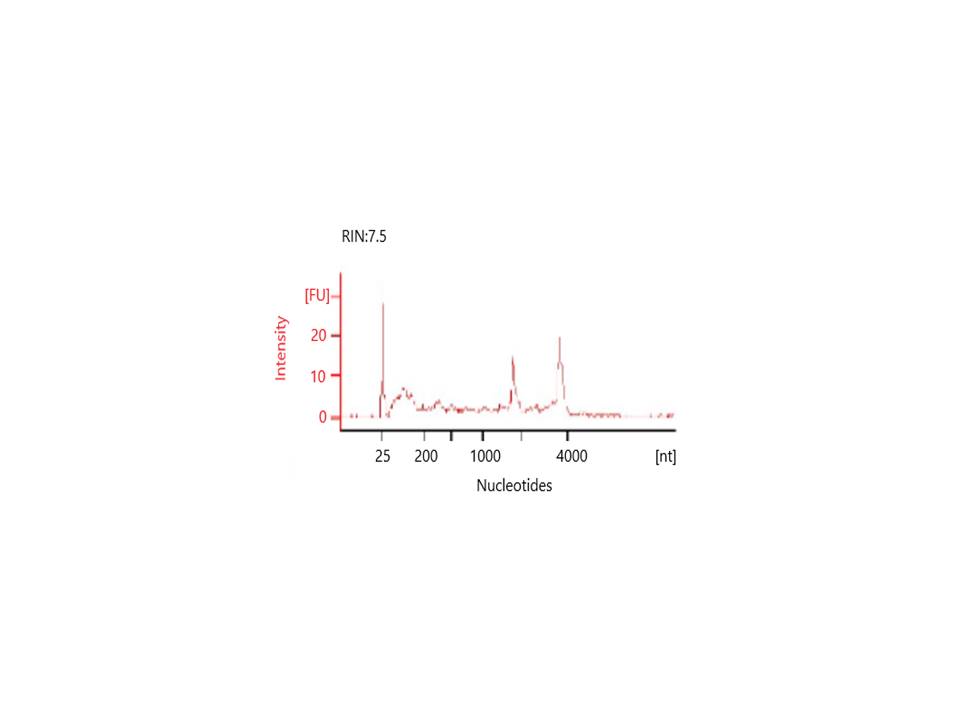

Supplement: Supplementary Figure 1 — The tissue contains RNA of good quality. [file Image_1.JPEG]

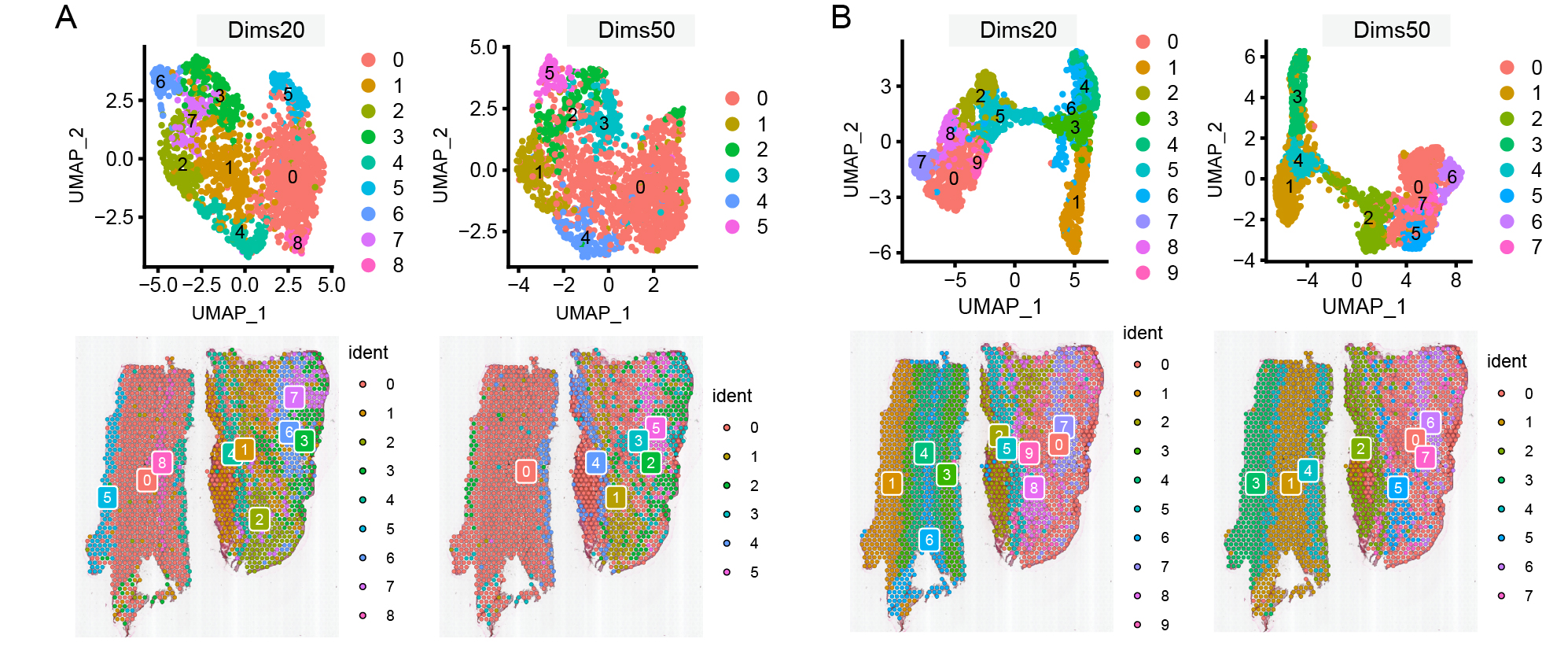

Supplement: Supplementary Figure 2 — Comparison of compositional analysis and dimensionality reduction. (A) The first row shows a comparison PCA of UMAP dim 20 and dim 50. The second row shows the UMIs distribution of each cluster, and the different clusters are labeled by using different colors. (B) The first row shows a comparison ICA of UMAP dim 20 and dim 50. The second row shows the UMIs distribution of each cluster, and different clusters are labeled using different colors. [file Image_2.JPEG]

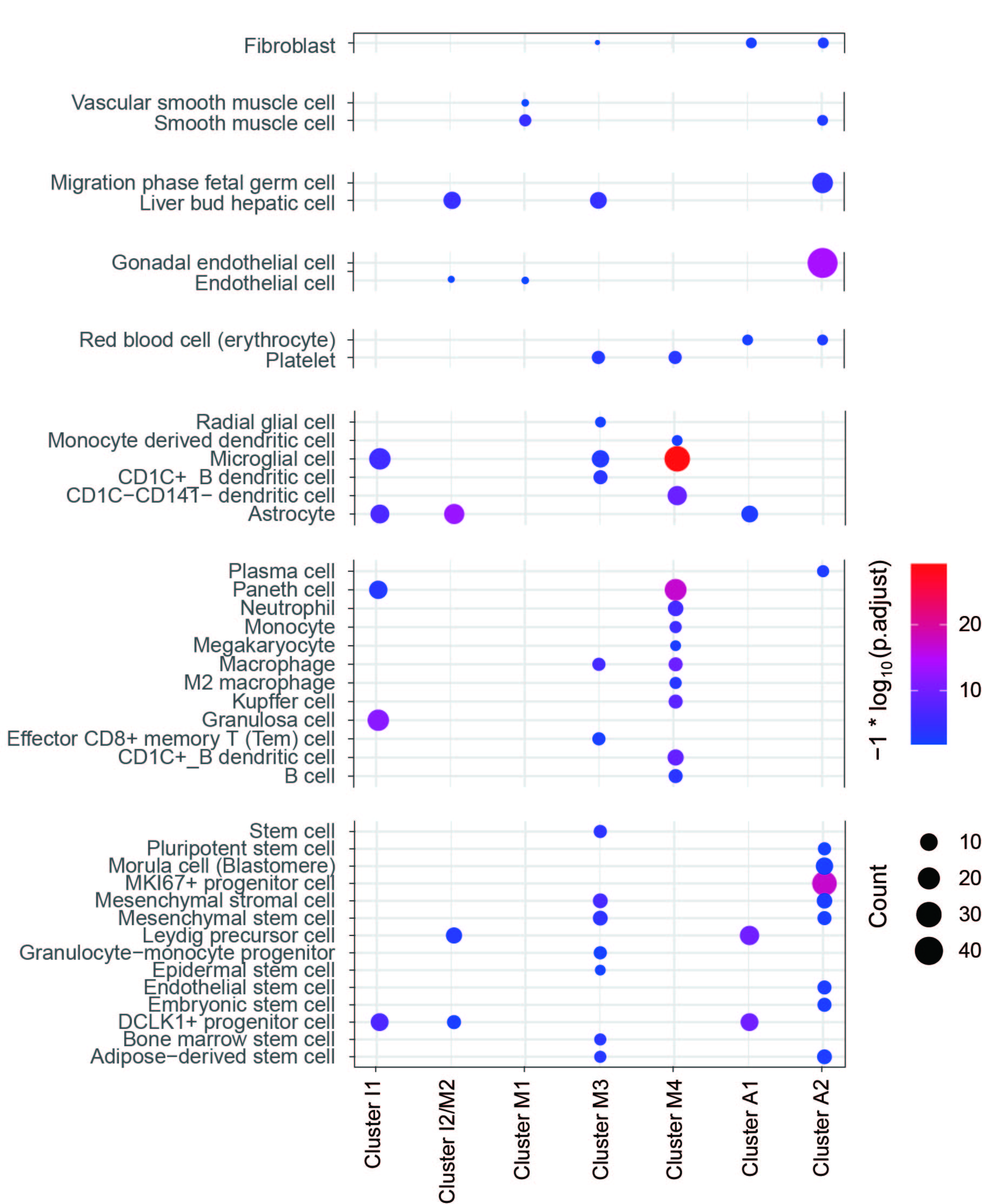

Supplement: Supplementary Figure 3 — Cell types identified by CellMarker database. The identified cell types in each cluster, which are annotated based on existed marker gene information. The gradient color represents the P-value; the size of the black spots represents the gene number. [file Image_3.JPEG]

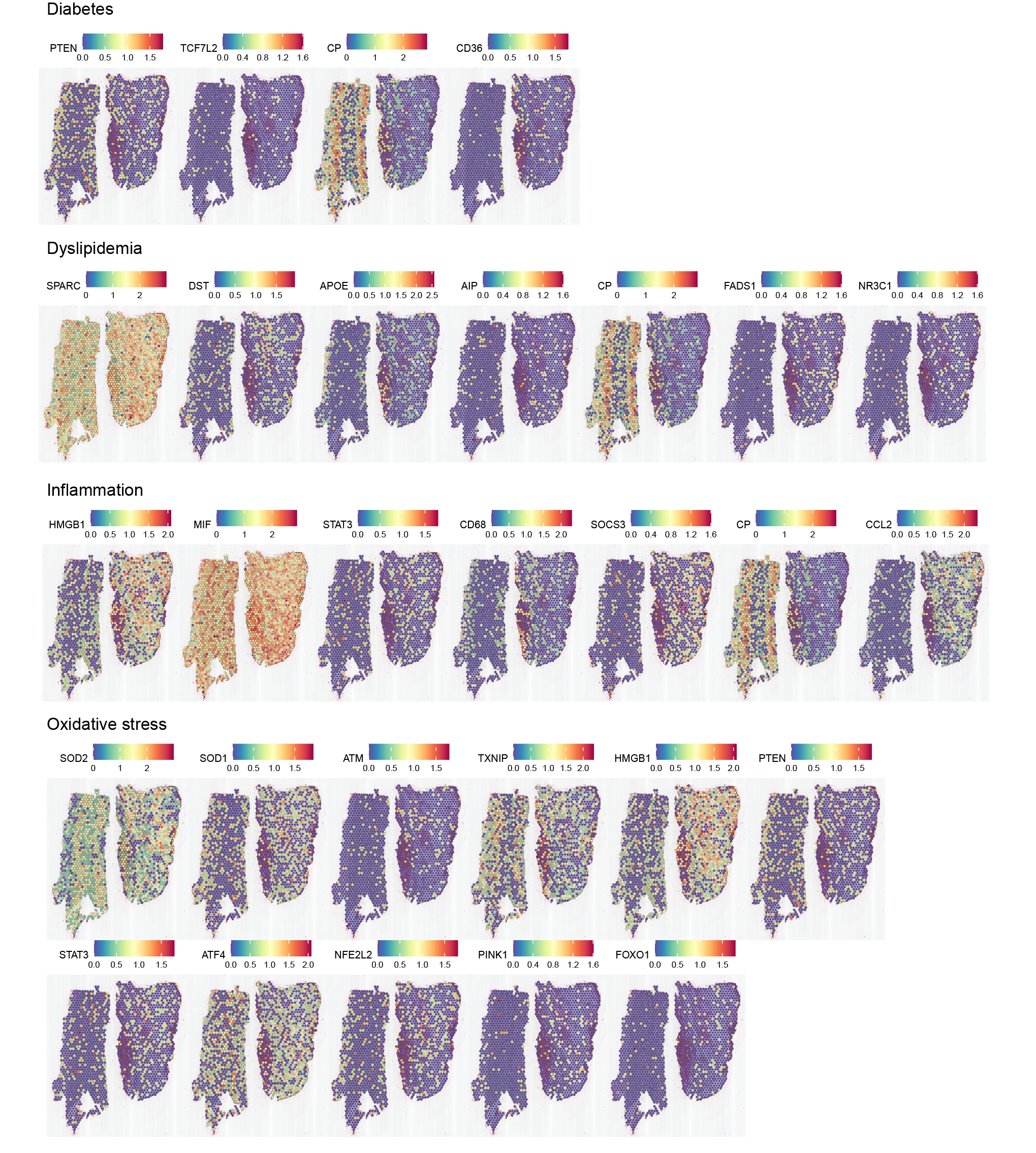

Supplement: Supplementary Figure 4 — Expression of highly expressed genes in dissection-related pathogenic factors in aortic dissection. Aortic dissection has reported the expression of highly expressed genes in dissection tissues of pathogenic factors (diabetes, dyslipidemia, inflammation, oxidative stress). These factors genes of ST profiles are listed. [file Image_4.JPEG]
