# Supplementary material for: Visualization and Analysis of Gene Expression in Stanford Type A Aortic Dissection Tissue Section by Spatial Transcriptomics
Source: Front Genet. 2021 Jun 28;12:698124. doi: 10.3389/fgene.2021.698124 (PMC8275070; doi:10.3389/fgene.2021.698124)
Supplement: Supplementary Table 1 — Basic information of patient. [file Table_1.DOCX]

**Table S1 Basic information of patients**

| Subject | characteristic | | Detail | | Space ranger summary | |
| --- | --- | --- | --- | --- | --- | --- |
| Individual 1 | Gender/Ages(years) | Male/50 | Resection of primary tear | Y | Number of Spots | 1873 |
|  | History of smoking | N | Ascending aorta replacement | Y | Number of Reads | 339,194,372 |
|  | History of drinking | N | Cardiopulmonary bypass time(min) | 173 | Valid Barcodes | 97.0% |
|  | Coronary artery disease | N | Hypothermic circulatory arrest | Y | Valid UMIs | 99.9% |
|  | Hypertension | Y | Retrograde cerebral perfusion | Y | Fraction Reads in Spots Under Tissue | 79.1% |
|  | Diabetes mellitus | N |  |  | Mean Reads per Spot | 181,097 |
|  | Atherosclerosis | N |  |  | Median Genes per Spot | 1,398 |
|  |  |  |  |  | Total Genes Detected | 19,879 |
